# Interdependency between corrections in the three anatomic planes in AIS instrumentation

**DOI:** 10.1186/1748-7161-10-S1-O22

**Published:** 2015-01-19

**Authors:** Xiaoyu Wang, Laure Boyer, Franck LeNaveaux, Hubert Labelle, Stefan Parent, Carl-Éric Aubin

**Affiliations:** 1 Polytechnique of Montreal, Montreal, QC, Canada; 2 Research Center, Sainte-Justine University Hospital Center, Montreal, QC, Canada

## Background and objective

In surgical instrumentation of scoliotic spine, correction maneuvers are performed with 3D correction principles to achieve deformity reduction in specific anatomic planes. The objective was to evaluate the interdependency between the effects of the correction maneuvers in the 3 anatomical planes during the correction process.

## Methods

A validated patient-specific biomechanical modeling and simulation technique was used to assess the 3D correction of a Lenke-1 AIS case through posterior spinal instrumentation. Uniplanar pedicle screws were bilaterally placed at the 2 proximal, 2 distal, and 3 apical levels. The simulation steps included only the concave side 5.5 mm Cobalt-Chrome rod attachment and its derotation followed by apical vertebral derotation. Eighteen instrumentation simulations were performed with different rod contours (curvatures of 10, 20 and 30 degrees), rod derotation (70, 90 and 110 degrees), and vertebral derotation torques (3 and 5 Nm per screw at the 3 apical levels). Indices in the 3 planes (Cobb angle, thoracic kyphosis, apical vertebral rotation (AVR)) were computed for each simulation.

## Results

For the eighteen simulations, the coronal plane correction through the concave side rod attachment and derotation was accompanied by an increase of the AVR from 10 to 15 degrees. The increase of thoracic kyphosis was proportional to the preoperative rod curvature and the rod derotation angle. AVR correction through apical vertebral derotation was accompanied by a slight loss of thoracic kyphosis and small improvement of coronal plane correction.

## Preliminary conclusion

the more the coronal Cobb angle was reduced through the concave side rod attachment and derotation, the more the AVR was worsened. The effect of the apical vertebral derotation on the coronal and sagittal planes was clinically not significant.

